# The Effect of Tolvaptan on Metabolism and Electrolyte Homeostasis in Patients with Heart Failure: A Systematic Review and Meta-Analysis

**DOI:** 10.31083/j.rcm2509334

**Published:** 2024-09-19

**Authors:** Yao Xiao, Yue Chen, Xianghao Zuo, Kadireya Mutalifu, Xiaoping Chen, Kai Liu

**Affiliations:** ^1^Department of Cardiology, West China Hospital, Sichuan University, 610041 Chengdu, Sichuan, China; ^2^Department of Clinical Medicine, West China Hospital, Sichuan University, 610041 Chengdu, Sichuan, China; ^3^Department of Pharmacy, West China Tianfu Hospital, Sichuan University, 610041 Chengdu, Sichuan, China; ^4^West China School of Medicine/West China Hospital, Sichuan University, 610041 Chengdu, Sichuan, China

**Keywords:** tolvaptan, heart failure, metabolism, meta-analysis

## Abstract

**Background:**

This study aimed to investigate the effect of tolvaptan on metabolism and electrolyte homeostasis in patients with heart failure (HF).

**Methods:**

Literature databases, such as PubMed, EMBASE, the Cochrane Library, China National Knowledge Infrastructure, VIP, and WanFang Data, were systematically searched for relevant trials from inception to November 4, 2023. We used the fixed effect model to combine the effect sizes and used *I^2^* to test heterogeneity. Funnel plots were plotted to assess publication bias.

**Results:**

16 studies were eligible for further analysis. No significant differences were identified in the incidence of hyperuricemia between the tolvaptan group and the placebo group (odds ratio (OR) = 1.23, 95% confidence interval (CI) = 0.97 to 1.55, *p* = 0.09). Tolvaptan decreased the levels of blood uric acid compared to traditional diuretics (mean difference (MD) = –82.8, 95% CI = –96.48 to –69.13, *p* < 0.00001). There was no significant difference in hypernatremia (OR = 1.62, 95% CI = 0.66 to 3.96, *p* = 0.29) and hyperkalemia (OR = 1.17, 95% CI = 0.93 to 1.48, *p* = 0.18) between the tolvaptan and control groups.

**Conclusions:**

Tolvaptan reduced the level of blood uric acid compared to conventional diuretics, and could be used as a substitute for traditional diuretics for HF patients with a high risk of gout.

## 1. Introduction

Heart failure (HF) has an adverse influence on a patients’ quality of life and life expectancy, especially when it results in lower extremity edema and dyspnea resulting from fluid retention. Patients with HF often use diuretics to decrease the symptoms of fluid overload, improve pulmonary congestion and alleviate dyspnea in the process of diagnosis and treatment [1]. However, loop and thiazide diuretics could lead to side effects including electrolyte and metabolic disorders [2, 3]. The increase in serum uric acid caused by conventional diuretics changes by 6%–21% from the corresponding baseline value, which especially loop diuretics can increase the risk of gout [4, 5]. Therefore, they were usually not used long-term or in sufficient doses in patients with high uric acid levels or gout. The adverse effects of loop and thiazide diuretics are associated with a worse prognosis in HF [6]. As stated in a meta-analysis, every 1 mg/dL increase in uric acid in HF patients would result in a 4% increase in all-cause mortality, and a 28% increase in major adverse events [6]. Therefore, it is necessary to find a substitute for traditional diuretics in patients with HF to minimize electrolyte disturbances and metabolic disorders.

Tolvaptan, as a selective vasopressin V2 receptor antagonist, binds to V2 receptors on collecting ducts and blocks its activity, causes aquaporin-2 (AQP2) to detach from the intima and lowers its expression, and prevents V2 receptor-mediated water reabsorption. It can exert a diuretic effect without affecting the absorption and excretion of electrolytes in this process [7]. When prescribing tolvaptan, it is essential to understand the adverse effects of tolvaptan compared with placebo or conventional diuretic therapy in HF patients. Numerous clinical studies have shown that the main adverse events of tolvaptan are dry mouth, thirst, dizziness, and urinary frequency. However, the effects of tolvaptan on the metabolism of uric acid, blood glucose, and blood lipids in HF patients remains unclear [8]. A review found that tolvaptan was associated with a 2.8 mEq/L–3.5 mEq/L rise in serum sodium levels [9], and that tolvaptan may cause electrolyte disturbances by affecting blood volume. However, few studies have reported the impact of tolvaptan on the incidence of hypernatremia and hyperkalemia. Therefore, we conducted this review and meta-analysis to quantitatively evaluate these adverse effects of tolvaptan in patients with HF.

## 2. Materials and Methods

### 2.1 Retrieval Strategy

This article was performed based on the Preferred Reporting Items for Systematic Reviews and Meta-analyses (PRISMA) statement (PRISMA 2020 checklist form) [10]. Two authors (YX and YC) independently searched pertinent articles through PubMed, EMBASE, the Cochrane Library, China National Knowledge Infrastructure (CNKI), VIP and WanFang databases from inception to November 4, 2023. In addition, the authors manually searched the references cited in potentially eligible items. The disagreements were resolved by consulting the third author (XHZ). The search keywords included tolvaptan and HF. The retrieval strategy for PubMed is listed in the supplementary document.

### 2.2 Inclusion and Exclusion Criteria

Studies were included if the following criteria were met: (1) randomized controlled trials (RCTs) or cohort studies published in Chinese or English; (2) patients with chronic heart failure or acute heart failure diagnosed based on the European Society of Cardiology (ESC) Guidelines [11]; (3) patients were divided into two groups, and patients were administered tolvaptan in intervention groups and they received treatment with control, placebo, or conventional diuretic agents in the control groups; and (4) objectives were in place to observe the incidence of adverse events, such as hyperuricemia (serum uric acid levels >7 mg/dL in males and >6 mg/dL in females) or increasing blood uric acid, elevated blood glucose or new-onset diabetes, elevated blood lipid or new-onset hyperlipidemia, hypernatremia (serum sodium level >145 mEq/L), and hyperkalemia (serum potassium level >5.5 mEq/L).

Studies were excluded if (1) there were other experimental concomitant medications (such as dopamine); (2) HF was not referred to as the primary diagnosis but as an accompanying symptom; (3) the subjects studied were patients who needed an assisted circulation device or dialysis; (4) there was no appropriate safety-related indicator in the studies; (5) they were case studies or literature reviews; or (6) studies of low quality or at high risk of bias.

### 2.3 Data Extraction

Two authors (YX and YC) independently reviewed articles by browsing the title, abstract and full text based on established inclusion and exclusion criteria. Discrepancies were resolved by discussion with the third author (XHZ). The extraction consisted of the two main parts (basic characteristics for each independent study and the outcomes). The basic information for each independent study included the author’s name, publication date, study design, types of control groups, tolvaptan dosages, sample size, follow-up, and study location. The primary outcomes were the rates of adverse events, such as hyperuricemia or increasing blood uric acid, elevated blood glucose or new-onset diabetes and elevated blood lipid or new-onset hyperlipidemia in tolvaptan and control groups. The secondary outcomes were the incidence of hypernatremia and hyperkalemia, and change in body weight in the tolvaptan and control groups. If the outcome data in the study were incomplete, only similar data was merged and analyzed in the final analysis. If the presentation of outcomes were different, such as means and standard deviations and medians and quartiles, these would be adjusted to be consistent using an algorithm.

### 2.4 Quality Assessment

Two researchers (YX and YC) independently assessed the quality and risk of bias of eligible studies, and disagreements were resolved through consultation with the third author (XHZ). The quality of the included cohort studies was evaluated using the Newcastle-Ottawa scale (NOS) [12]. An overall score of NOS is ten and studies enrolled with a score of less than seven were excluded. The quality and risk of bias of the included RCTs were assessed based on the Cochrane Collaboration’s Risk of Bias (RoB) [13]. This assessment tool includes six items, and studies enrolled which had no clear description of the six items were considered to be of high risk of bias and were excluded.

### 2.5 Statistical Analysis

RevMan 5.3 software (Copenhagen: The Nordic Cochrane Centre, The Cochrane Collaboration, Denmark) was used to perform the statistical analysis. Dichotomous variables were analyzed as frequency and proportion, with odds ratio (OR) and corresponding 95% confidence interval (CI) for the assessment of outcome effects. Continuous data were reported by using mean difference (MD) and its 95% CI [14]. The *I*-square test was used to test heterogeneity among studies, and the fixed effect model (*I*^2^ < 50%) or the random effect model (*I*^2^ ≥ 50%) was selected to combine the effect sizes and 95% CI across studies. Subgroup analysis and meta-regression analysis were used to explore the potential sources of heterogeneity if necessary. Sensitivity analysis was performed to assess how stable the findings were and to evaluate the influence of individual studies on the outcomes. Publication bias was investigated with a funnel plot. A two-tailed *p* < 0.05 was considered statistically significant.

## 3. Results

### 3.1 Study Selection

**Fig. 1.**
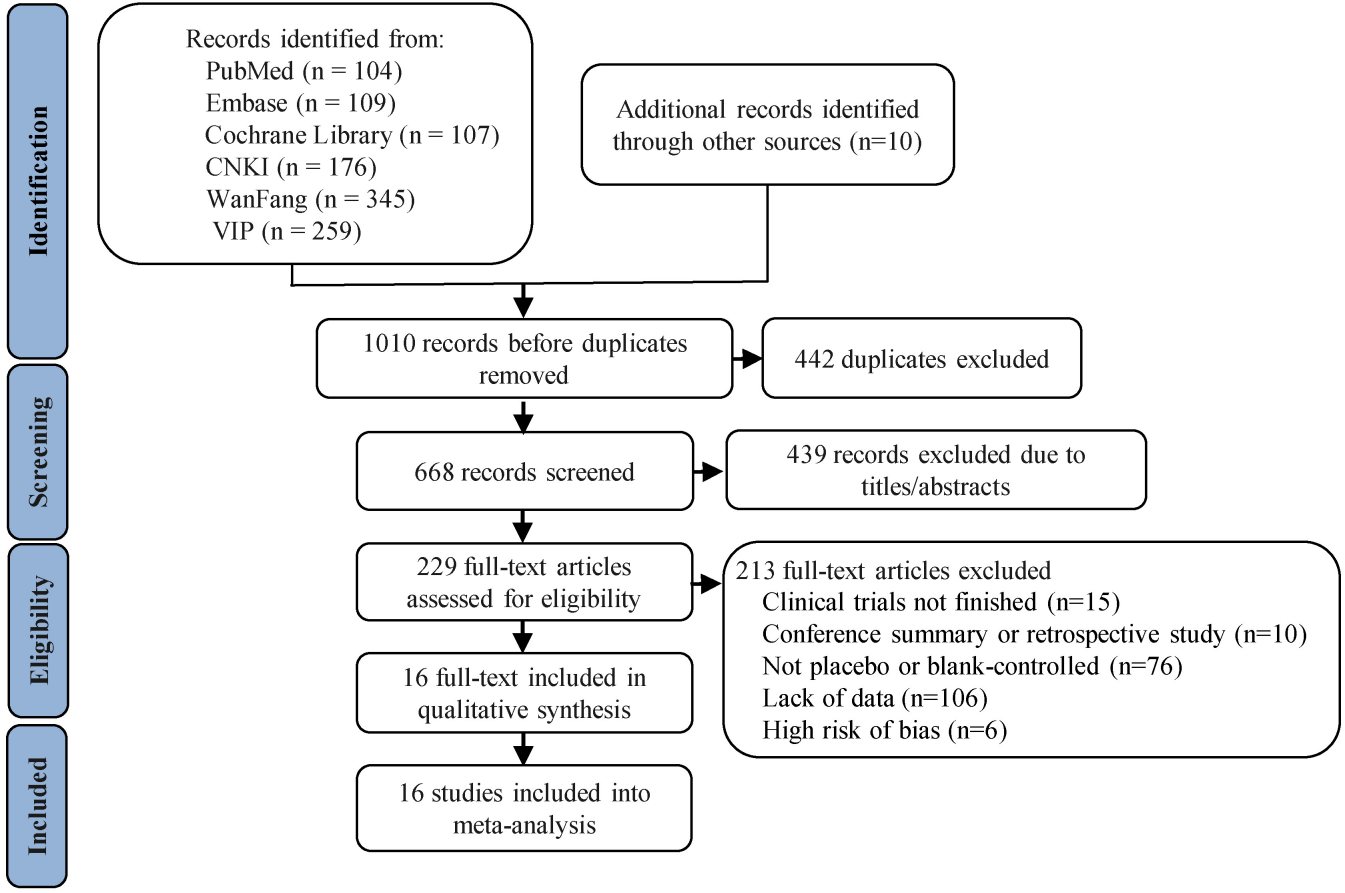
**Flow chart of the literature screening process**.

A summary of the search and screening process is shown in Fig. 1. After searching the databases according to the retrieval strategy, 1100 studies were identified in total, and 10 articles were supplemented through screening the reference lists of the included articles. After duplicate removal, 668 studies remained and were screened for potential eligibility. Of them, 229 studies were retained for full text evaluation, and 213 were excluded for assorted reasons (see Fig. 1 for details). Finally, 16 articles were enrolled in the final meta-analysis.

### 3.2 Characteristics of Included Studies

The detailed characteristics of the 16 studies included are shown in Table 1 [15, 16, 17, 18, 19, 20, 21, 22, 23, 24, 25, 26, 27, 28, 29, 30] and **Supplementary Table 1**. The publication date of the enrolled studies ranged from 2005 to 2023, and 5949 patients were involved in our study in total. Of the 16 articles, nine were in Chinese [22, 23, 24, 25, 26, 27, 28, 29, 30], and the remaining were in English [15, 16, 17, 18, 19, 20, 21]. The follow-up duration across different studies ranged from two days to one year, and the average duration of follow-up was 31 days.

**Table 1.** **Basic characteristics of enrolled studies**.

Author, Year	Study type	Trial design	Intervention		Sample size	Follow-up	Study location
			Tolvaptan	Control			
Katsuhisa Saito [15], 2005	Randomized	double-blind, placebo-controlled	OPC-41061 15–45 mg/d	Placebo	122	7 d	Asia
Katsuhisa Saito [16], 2007	Randomized	double-blind, placebo-controlled	OPC-41061 15 mg/d	Placebo	110	7 d	Asia
Felker GM *et al*. [17], 2017	Randomized	double-blind, placebo-controlled	Tolvaptan 30 mg/d	Placebo	257	2 d	America
Gheorghiade M *et al*. [18], 2003	Randomized	double-blind, placebo-controlled	Tolvaptan 30–60 mg/d	Placebo	254	25 d	America
Komiya S *et al*. [19], 2022	Randomized	open-label, controlled	Tolvaptan 15 mg/d + Furosemide	Furosemide	33	7 d	Asia
Konstam MA *et al*. [20], 2007	Randomized	double-blind, placebo-controlled	Tolvaptan 30 mg/d	Placebo	4133	9.9 m	18 countries
Shanmugam E *et al*. [21], 2016	Randomized	double-blind, placebo-controlled	Tolvaptan 15 mg/d	Placebo	51	5 d	Asia
Cheng AY *et al*. [22], 2023	Randomized	single-blind, controlled	Tolvaptan 7.5–30 mg/d + Conventional diuretics	Conventional diuretics	60	3 m	Asia
Peng YL *et al*. [23], 2018	Randomized	controlled	Tolvaptan 15 mg/d + Conventional diuretics	Conventional diuretics	60	7 d	Asia
Clinical Study Group of Tolvaptan [24], 2017	Randomized	double-blind, placebo-controlled	Tolvaptan 15 mg/d	Placebo	244	7 d	Asia
Wang L *et al*. [25], 2023	Randomized	controlled	Tolvaptan 15 mg/d + Conventional diuretics	Conventional diuretics	76	7 d	Asia
Ye TQ *et al*. [26], 2020	Randomized	controlled	Tolvaptan 7.5–15 mg/d + Conventional diuretics	Conventional diuretics	110	1 m	Asia
Zhang D *et al*. [27], 2016	Randomized	controlled	Tolvaptan 15 mg/d + Conventional diuretics	Conventional diuretics	54	7 d	Asia
Cui ZT *et al*. [28], 2023	Randomized	controlled	Tolvaptan 7.5 mg/d + Furosemide	Furosemide	127	7 d	Asia
Ren B *et al*. [29], 2019	Randomized	controlled	Tolvaptan 7.5–15 mg/d + Conventional diuretics	Conventional diuretics	60	8 d	Asia
Cai JH *et al*. [30], 2023	Retrospective cohort	controlled	Tolvaptan + Conventional diuretics	Conventional diuretics	198	6 m	Asia

Footnote: OPC-41061, synonym of tolvaptan.

### 3.3 Risk of Bias Assessment

Assessment of the quality and risk of bias for the included studies is outlined in **Supplementary Fig. 1** and **Supplementary Table 2**. Both the included retrospective cohort study and RCTs were of moderate to high quality.

### 3.4 Hyperuricemia or Increasing Blood Uric Acid

**Fig. 2.**
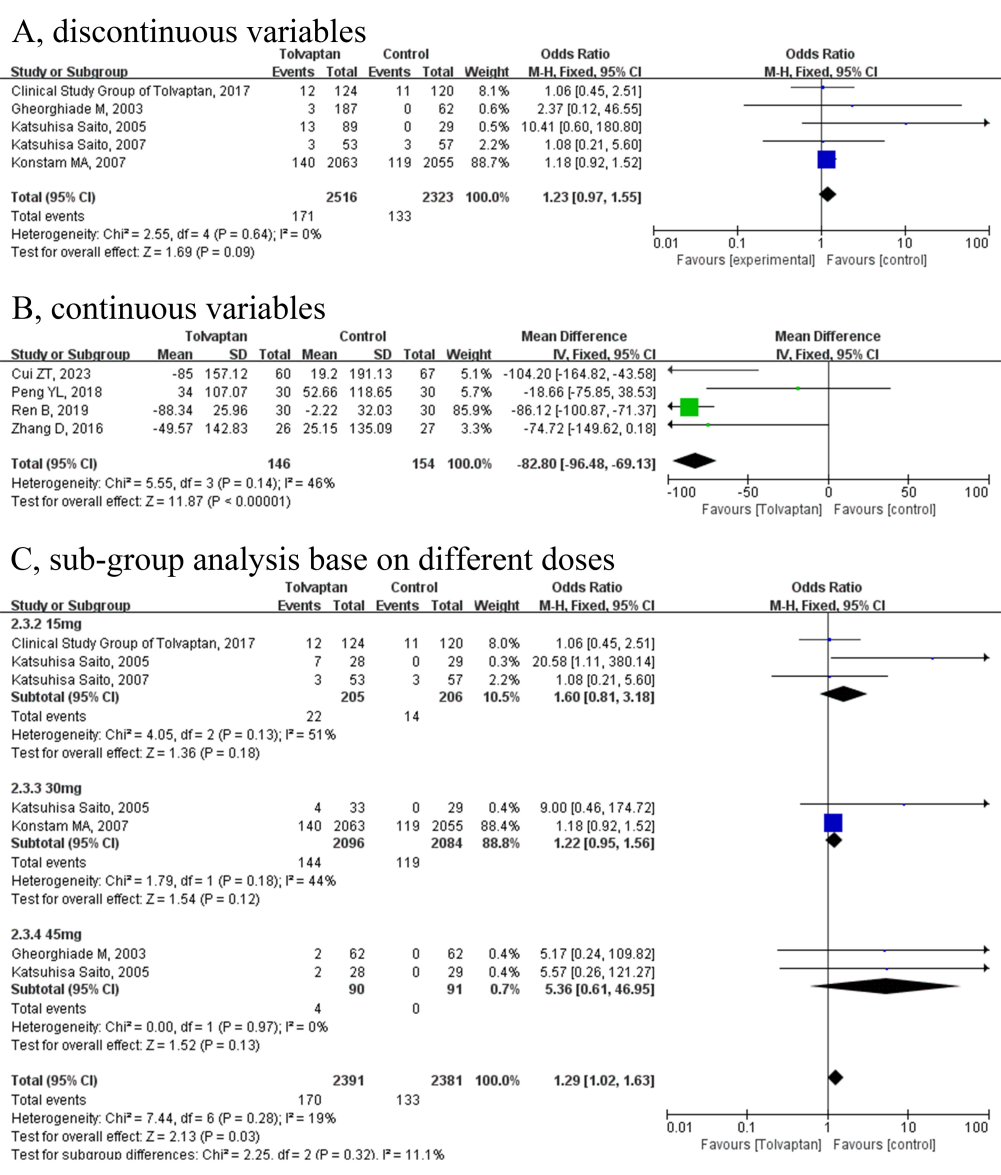
**Forest plots depicting the effects of tolvaptan on the incidence of hyperuricemia or increasing blood uric acid**. (A) discontinuous variable; (B) continuous variable; (C) sub-group analysis based on different doses. CI, confidence interval; M-H, Mantel-Haenszel; IV, inverse variance; SD, standard deviation.

In total, nine studies [15, 16, 18, 20, 23, 24, 27, 28, 29], involving 5139 patients for quantitative analysis, reported the incidence of hyperuricemia or increasing blood uric acid. The pooled data demonstrated that no significant difference was identified in the incidence of hyperuricemia between the tolvaptan group and the placebo group (OR = 1.23, 95% CI = 0.97 to 1.55, *p* = 0.09; Fig. 2A) while tolvaptan could decrease uric acid levels compared to the conventional diuretics group (MD = –82.8, 95% CI = –96.48 to –69.13, *p* < 0.00001; Fig. 2B) without statistical heterogeneity (*I*^2^ = 0%, *p* = 0.64; *I*^2^ = 46%, *p* = 0.14). It was shown in the subgroup analysis that different tolvaptan doses had no impact on the pooling results (Fig. 2C). The sample size of Konstam *et al*. [20] exceeded 80% of the total sample, and we conducted a sensitivity analysis to avoid potential bias. After excluding the article, the outcome suggested that the results were stable (**Supplementary Fig. 2**).

### 3.5 Other Metabolic Indicators: Blood Glucose and Lipids

In our study, two articles described the effect of tolvaptan on blood glucose [15, 16], and no study reported the effect of tolvaptan on blood lipids or the risk of new-onset diabetes or new-onset hyperlipidemia. One article published in 2005 reported that two patients with elevated blood glucose and two patients with hypoglycemia were found in the 15 mg/d tolvaptan group (2/28; 2/28), compared with no patients with elevated blood glucose or hypoglycemia in the placebo group (0/29; 0/29) [15]. The other article published in 2007 showed that hypoglycemia occurred in two patients taking placebo (2/57), compared with none in patients taking 15 mg/d tolvaptan (0/53) [16].

### 3.6 Electrolyte Disorders

**Fig. 3.**
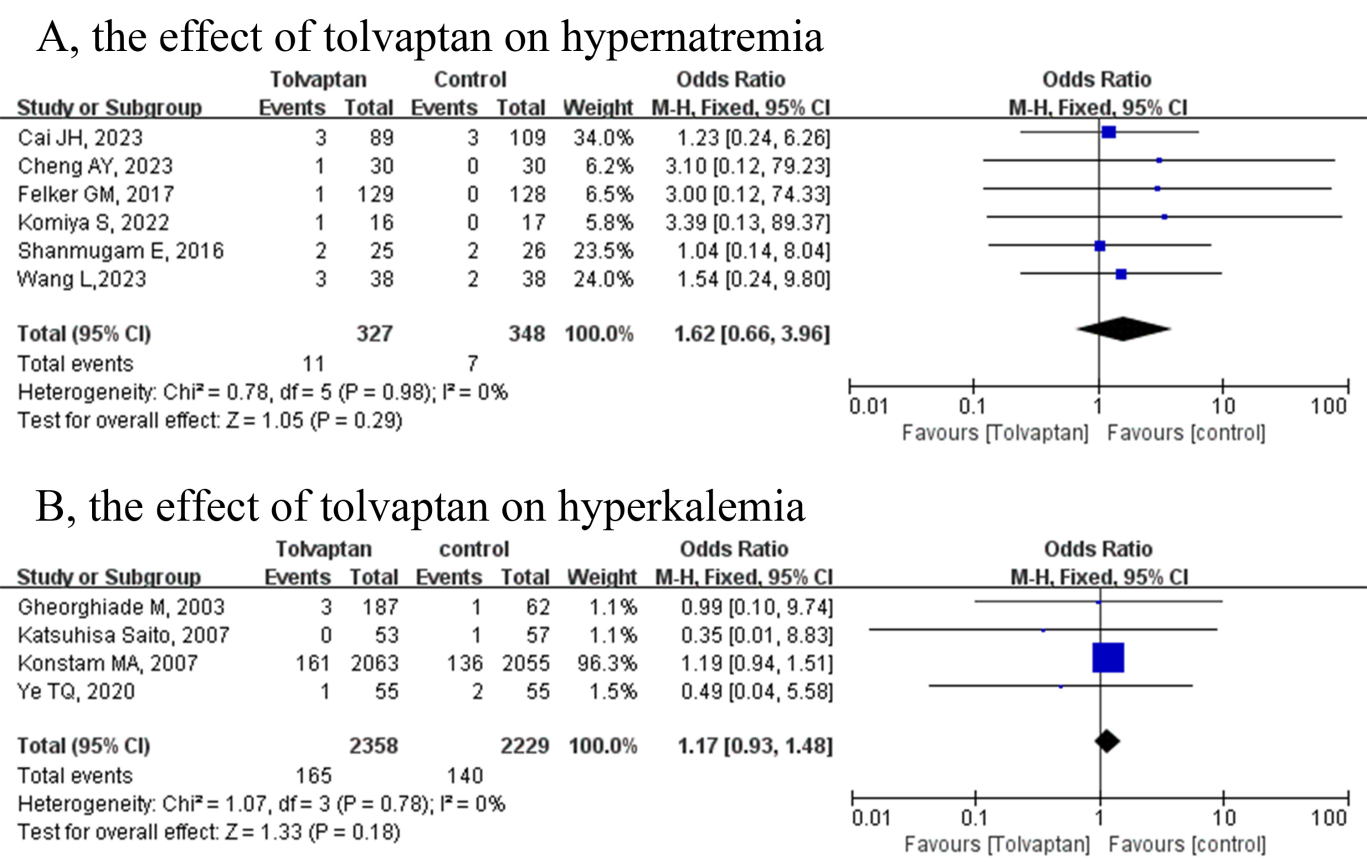
**Forest plots depicting the effects of tolvaptan on electrolytes**. (A) the effects of tolvaptan on hypernatremia; (B) the effects of tolvaptan on hyperkalemia. CI, confidence interval; M-H, Mantel-Haenszel.

In the 16 included studies [15, 16, 17, 18, 19, 20, 21, 22, 23, 24, 25, 26, 27, 28, 29, 30], six reported the risk of hypernatremia [17, 19, 21, 22, 25, 30], while four reported the risk of hyperkalemia [16, 18, 20, 26]. As shown in Fig. 3, there was no significant difference regarding hypernatremia (OR = 1.62, 95% CI = 0.66 to 3.96, *p* = 0.29) and hyperkalemia (OR = 1.17, 95% CI = 0.93 to 1.48, *p* = 0.18) between the tolvaptan and control groups without statistical heterogeneity (*I*^2^ = 0%, *p* = 0.98; *I*^2^ = 0%, *p* = 0.78). After excluding the study by Konstam *et al*. [20], the results were still stable (**Supplementary Fig. 3**).

### 3.7 Diuretic Effects’ Indicators: Change in Body Weight

**Fig. 4.**
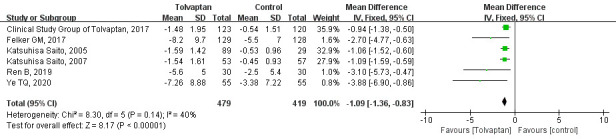
**Forest plots depicting the effects of tolvaptan on the change in body weight during follow-up**. SD, standard deviation; CI, confidence interval; IV, inverse variance.

Six studies reported the change in body weight during follow-up [15, 16, 17, 24, 26, 29], with five reporting short-term effects (<1 m) [15, 16, 17, 24, 29] and one reporting long-term effects (≥1 m) [26]. As shown in Fig. 4, the data demonstrated that tolvaptan led to greater weight loss than control group (MD = –1.09, 95% CI = –1.36 to –0.83, *p* < 0.00001) without statistical heterogeneity (*I*^2^ = 40%, *p* = 0.14).

### 3.8 Publication Bias

To determine publication bias, we conducted funnel plots comparing the effects of tolvaptan on the incidence of hyperuricemia or changes in blood uric acid. The results showed no obvious publication bias in the analysis (**Supplementary Fig. 4**).

## 4. Discussion

This is the first meta-analysis involving the effect of tolvaptan on metabolism in patients with HF. Our study showed that tolvaptan reduced blood uric acid level compared with traditional diuretics while it exerted no apparent impact on the incidence of hyperuricemia in patients with HF compared to placebo. There were insufficient data to support the analysis of the effect of tolvaptan on blood glucose and lipids. The results demonstrated that no significant difference was identified between tolvaptan and control groups regarding electrolyte disturbances. Tolvaptan resulted in greater weight loss compared to the control group.

Patients with HF are usually admitted to the hospital because of increasing signs and symptoms caused by volume load, such as lower extremity edema and dyspnea [31]. Therefore, the use of diuretics is essential to decrease fluid retention and alleviate the signs and symptoms in HF patients. Traditional diuretics include loop and thiazide diuretics. Unlike the diuretic mechanism of tolvaptan, loop diuretics exert a diuretic effect by antagonizing the sodium/potassium/chloride cotransporter (NKCC2) in the thick segment of the ascending branch of the renal medullary loop [32], while thiazide diuretics inhibit the sodium/chloride cotransporter (NCC) in the distal convoluted tubule and prevent the reabsorption of sodium chloride as well as water at the same time [32]. These drugs have a significant effect on electrolyte disturbances especially sodium and potassium [1], and uric acid metabolism [33, 34, 35]. These adverse effects, in particular hyperuricemia, dramatically increase the risk of adverse events in HF patients [6].

High uric acid levels or hyperuricemia is common in patients with HF. Uric acid is the final product of purine nucleotide catabolism and xanthine oxidoreductase enzyme (XOR) is a rate-limiting enzyme of purine metabolism [36]. Hypoxia, acidosis, tissue hypoxia-ischemia and ischemia/reperfusion circulation can increase XOR activity [37, 38]. Uric acid is a macromolecule, which needs to be transported on the cell membrane through specific transporters. There are several uric acid transporters in the kidney and intestine, and approximately 70% of uric acid is secreted through the kidneys [39]. In HF, especially in the acute progression of HF, tissue ischemia and hypoxia will cause the XOR activity and catabolic pathway up-regulation, purine degradation increasing and even renal excretion damage, both lead to the rise in uric acid levels [40, 41, 42].

In theory, loop and thiazide diuretics could increase the reabsorption of blood uric acid at the lateral base of the proximal renal tubules [43, 44]. These two kinds of diuretics also induce the secretion of uric acid by antagonizing the human sodium phosphate transporter 4 (NPT4), which functions as a voltage-driven urate transporter [43]. Furthermore, they inhibit multidrug resistance associated protein 4 (MRP4) which functions as a luminal efflux transporter for urate in the proximal tubule, which may also cause hyperuricemia [45]. Long term use of traditional diuretics would lead to further deterioration of renal function and affect the excretion of uric acid [46].

There is no evidence that tolvaptan affects uric acid production, excretion, or reabsorption. Previous study has showed that tolvaptan could prevent the deterioration of renal function [47], which may be beneficial for the metabolism of uric acid.

In previous studies comparing tolvaptan and different traditional diuretics, tolvaptan significantly reduced blood uric acid levels in patients with HF compared with furosemide [48], whereas the effects of tolvaptan and hydrochlorothiazide on patients’ uric acid levels were similar [49]. This may be due to the relatively small number of studies with small sample sizes [48, 49]. Therefore, more RCTs are required to further study this effect of tolvaptan.

In addition, conventional diuretics cause elevated blood glucose and insulin resistance [50, 51], which may be mediated by hypokalemia and hyperuricemia [52, 53, 54, 55]. Our study indicated that tolvaptan had no impact on the incidence of hyperuricemia. Tolvaptan can increase the excretion of water and concentrates the blood, which may cause an increase in serum potassium [7]. So tolvaptan cannot lead to hyperglycemia by this pathway in theory. Some studies showed that thiazide-type diuretics could increase sympathetic nerve activity [56, 57]. The sympathetic nervous system plays an essential role in promoting insulin resistance by reducing glucose uptake in skeletal muscle [58, 59], which could increase blood glucose. Compared with traditional diuretics, tolvaptan significantly reduced sympathetic nerve system activation [60]. Thus, tolvaptan may be superior to traditional diuretics in maintaining blood glucose levels theoretically. The included trials in our study revealed that no significant difference was observed in the incidence of hyperglycemia or hypoglycemia between tolvaptan and the placebo group [15, 16]. Besides, traditional diuretics, especially hydrochlorothiazide, may cause dyslipidemia, such as elevated levels of total cholesterol and triglycerides [61, 62]. Hydrochlorothiazide interferes with normal fat metabolism by activating the renin-angiotensin-aldosterone system, increasing C-reactive protein and plasminogen activator inhibitors, causing mild inflammation, promoting inflammation of adipose tissue and secretion of inflammatory mediators into the blood circulation, reducing adiponectin, increasing the incidence of inflammatory adipose tissue and the secretion of inflammatory mediators into the circulation, causing a mild systemic inflammatory response, and increasing blood lipid levels [63]. However, no studies have been performed to investigate the effect of tolvaptan on blood lipid metabolism. The study conducted by Zhang [49] demonstrated that there was no difference between tolvaptan and hydrochlorothiazide on the effect on blood glucose and blood lipid levels. However, the sample size of this study was small [49]. Therefore, more RCTs with a larger sample size are still needed to evaluate the effect of tolvaptan on glycolipid metabolism.

The effect of tolvaptan is to increase the rate of removal of free water by inhibiting the reabsorption of water in the renal collecting duct, which may cause an increase in Na+ concentration [64]. In theory, the incidence of hypernatremia was higher in the tolvaptan group than in the control group. However, in our meta-analysis, no significant difference was identified between the two groups regarding electrolyte disturbance, which was similar to previous study [65]. The pharmacokinetics of tolvaptan indicate that the effect of diuresis and elevation of blood sodium concentration begins within approximately 2 to 4 hours after administration, and peaks at 4 to 8 hours. 24 hours after administration, 60% of the sodium ions still remain at the peak concentration [66]. The SALT-1 and SALT-2 clinical trials also demonstrated that tolvaptan could increase the serum Na+ concentration [67]. The mechanism of action of tolvaptan was consistent with the results of other clinical trials. The study by Gunderson EG *et al*. [9] demonstrated that tolvaptan increased serum sodium concentrations by an average of 2.8 mEq/L to 3.5 mEq/L in the first 24 hours. Therefore, an explanation for our results may be that the increase in serum sodium plus serum sodium at baseline in each study did not reach hypernatremia levels, which may be similar to the incidence of hyperkalemia in our study.

There are numerous RCTs and meta-analyses investigating the diuretic effect of tolvaptan, mainly the short-term effect [17, 68, 69]. Change in body weight and urine output are commonly used in this field. This study assessed change in body weight to reflect the diuretic effect and hoped to further explore the difference between the longterm and short-term effect. The data indicated that tolvaptan caused a greater change in body weight from baseline compared to the control group, which was consistent with previous literature [17, 68, 69]. As there was only an included study in the subgroup of long-term effect [26], we were unable to perform a subgroup analysis based on the duration of follow-up to investigate the long-term or short-term diuretic effects of tolvaptan. Therefore, a meta-analysis focusing on short-term and long-term diuretic effect can be performed in the future.

There are several limitations in our study. First, the study design of some included trials was single blind or open label and the proportion of Chinese articles in the included articles was high, which might lead to bias. But, overall, the studies included were of moderate to high quality. Second, in the meta-analysis of uric acid, the sample sizes varied across the studies included, which may lead to inaccuracy of the pooled results despite the null heterogeneity found in the merger analyses. Besides, only one trial reporting the level of serum uric acid had a long-term follow-up (9.9 months), so we could not conduct a subgroup analysis to explore the short-term or long-term effect of tolvaptan on the level of serum uric acid. Long-term effect of tolvaptan on serum uric acid needs to be further studied. Third, in the subgroup analysis, the number of studies and the sample size of most dose groups were small and increasing the sample size would make the results more accurate and reliable. Fourth, since the original studies did not provide data on gender, age, and dose for the relevant subgroups, we were unable to perform subgroup analyses to further understand the effect of these factors on the outcomes. Fifth, we were not able to evaluate the effect of tolvaptan on glycolipid metabolism due to the lack of related original studies. In general, more evidence is required to focus on the adverse effects of tolvaptan, particularly its effects on metabolism. Finally, as our study focused on the adverse effects of tolvaptan, the outcomes did not include indicators of diuretic effect in the inclusion criteria, which may lead to the absence of articles related to diuretic effect and ultimately caused the failure of subgroup analyses.

## 5. Conclusions

Compared with traditional diuretics, tolvaptan reduced the level of blood uric acid, which is beneficial to the prognosis of patients with HF. Tolvaptan could also be used as an alternative to traditional diuretics for HF patients with high risk of gout. The effect of tolvaptan on the metabolism of blood glucose and blood lipids is still unclear and needs to be further studied.

## Availability of Data and Materials

The datasets used and/or analyzed during the current study are available from the corresponding author on reasonable request.

## Abbreviations

HF, heart failure; AQP2, aquaporin-2; PRISMA, preferred reporting items for systematic reviews and meta-analyses; CNKI, China national knowledge infrastructure; RCTs, randomized controlled trials; ESC, the European Society of Cardiology; NOS, Newcastle-Ottawa scale; RoB, cochrane collaboration’s risk of bias; CI, confidence interval; MD, mean difference; NKCC2, sodium/potassium/chloride cotransporter; NCC, sodium/chloride cotransporter; XOR, xanthine oxidoreductase enzyme; NPT4, human sodium phosphate transporter 4; MRP4, multidrug resistance associated protein 4.

## Supplementary Material

Supplementary material associated with this article can be found, in the online version, at https://doi.org/10.31083/j.rcm2509334.

## References

[1] Mullens W, Damman K, Harjola VP, Mebazaa A, Brunner-La Rocca HP, Martens P (2019). The use of diuretics in heart failure with congestion - a position statement from the Heart Failure Association of the European Society of Cardiology. *European Journal of Heart Failure*.

[2] Peacock WF, Costanzo MR, De Marco T, Lopatin M, Wynne J, Mills RM (2009). Impact of intravenous loop diuretics on outcomes of patients hospitalized with acute decompensated heart failure: insights from the ADHERE registry. *Cardiology*.

[3] Wilcox CS (1999). Metabolic and adverse effects of diuretics. *Seminars in Nephrology*.

[4] Reyes AJ (2003). Cardiovascular drugs and serum uric acid. *Cardiovascular Drugs and Therapy*.

[5] Bruderer S, Bodmer M, Jick SS, Meier CR (2014). Use of diuretics and risk of incident gout: a population-based case-control study. *Arthritis & Rheumatology (Hoboken, N*.

[6] Miao L, Guo M, Pan D, Chen P, Chen Z, Gao J (2021). Serum Uric Acid and Risk of Chronic Heart Failure: A Systematic Review and Meta-Analysis. *Frontiers in Medicine*.

[7] Hirano T, Yamamura Y, Nakamura S, Onogawa T, Mori T (2000). Effects of the V(2)-receptor antagonist OPC-41061 and the loop diuretic furosemide alone and in combination in rats. *The Journal of Pharmacology and Experimental Therapeutics*.

[8] Li B, Fang D, Qian C, Feng H, Wang Y (2017). The Efficacy and Safety of Tolvaptan in Patients with Hyponatremia: A Meta-Analysis of Randomized Controlled Trials. *Clinical Drug Investigation*.

[9] Gunderson EG, Lillyblad MP, Fine M, Vardeny O, Berei TJ (2019). Tolvaptan for Volume Management in Heart Failure. *Pharmacotherapy*.

[10] Moher D, Liberati A, Tetzlaff J, Altman DG, PRISMA Group (2009). Preferred reporting items for systematic reviews and meta-analyses: the PRISMA statement. *PLoS Medicine*.

[11] McDonagh TA, Metra M, Adamo M, Gardner RS, Baumbach A, Böhm M (2021). 2021 ESC Guidelines for the diagnosis and treatment of acute and chronic heart failure. *European Heart Journal*.

[12] Wells G, Shea B, O’Connell D, Robertson J, Peterson J, Welch V (2014). The Newcastle-Ottawa Scale (NOS) for assessing the quality of nonrandomised studies in meta-analyses. *Ottawa Hospital Research Institute*.

[13] Higgins JPT, Altman DG, Sterne JAC, Higgins Jpt, Green S (2011). Assessing risk of bias in included studies. *Cochrane handbook for systematic reviews of interventions, version 5.1.0*.

[14] Higgins JPT, Thompson SG, Deeks JJ, Altman DG (2003). Measuring inconsistency in meta-analyses. *BMJ*.

[15] Katsuhisa Saito (2005). A Dose-finding Study of OPC-41061 in Treatment of Cardiac Edema (Congestive Heart Failure). https://clinicaltrials.gov/study/NCT00234104?term=NCT00234104&rank=1.

[16] Katsuhisa Saito (2007). A Double-blind, Placebo-controlled Study of OPC-41061 in the Treatment of Cardiac Edema (Congestive Heart Failure). https://clinicaltrials.gov/study/NCT00462670?term=NCT00462670&rank=1.

[17] Felker GM, Mentz RJ, Cole RT, Adams KF, Egnaczyk GF, Fiuzat M (2017). Efficacy and Safety of Tolvaptan in Patients Hospitalized With Acute Heart Failure. *Journal of the American College of Cardiology*.

[18] Gheorghiade M, Niazi I, Ouyang J, Czerwiec F, Kambayashi JI, Zampino M (2003). Vasopressin V2-receptor blockade with tolvaptan in patients with chronic heart failure: results from a double-blind, randomized trial. *Circulation*.

[19] Komiya S, Katsumata M, Ozawa M, Haze T, Kawano R, Ohki Y (2022). Efficacy of tolvaptan on advanced chronic kidney disease with heart failure: a randomized controlled trial. *Clinical and Experimental Nephrology*.

[20] Konstam MA, Gheorghiade M, Burnett JC, Grinfeld L, Maggioni AP, Swedberg K (2007). Effects of oral tolvaptan in patients hospitalized for worsening heart failure: the EVEREST Outcome Trial. *JAMA*.

[21] Shanmugam E, Doss CRMP, George M, Jena A, Rajaram M, Ramaraj B (2016). Effect of tolvaptan on acute heart failure with hyponatremia–a randomized, double blind, controlled clinical trial. *Indian Heart Journal*.

[22] Cheng AY, Zhu CJ, Liu LY, He Y, Wang H, Xia CH (2023). Efficacy observation of tolvaptan in patients with acute reduced ejection fraction heart failure combined with renal dysfunction. *Chinese Heart Journal*.

[23] Peng YL, Huang J, Ma DX (2018). Effect of tolvaptan on patients with chronic heart failure. *Medical Journal of the Chinese People’s Armed Police Force*.

[24] Clinical Study Group of Tolvaptan (2017). A randomized, double-blind, placebo-controlled, multicenter study to assess the efficacy and safety of tolvaptan in heart failure patients with volume overload on the standard treatment with conventional diuretics. *Chinese Journal of Heart Failure and Cardiomyopathy*.

[25] Wang L, Yao Y, Zhang YY (2023). Effect of tolvaptan on the level of myocardial injury markers in patients with chronic heart failure complicated with hyponatremia. *Journal of Chinese Physician*.

[26] Ye TQ, Zhao L, Yu H, Gong Q, Zheng X (2020). Study on Tolvaptan in the Treatment of Heart Failure with Renal Insufficiency. *Medical Information*.

[27] Zhang D, Tan H, Li YH, Wang RR (2016). Efficacy and satefy of tolvaptan in treating heart failure. *China Medicine*.

[28] Cui ZT, Lu L, Guo QX, Liu JP (2023). Curative effect of V2 receptor antagonist combined with routine diuretics in patients with deep sternal wound infection complicated by heart failure coronary artery bypass grafting. *Chinese Journal of Evidence-Based Cardiovascular Medicine*.

[29] Ren B, Chen C, Guo YJ (2019). Efficacy and safety of tolvaptan in the treatment of elderly patients with chronic heart failure and diuretic resistance. *Chinese Journal of Prevention and Control of Chronic Diseases*.

[30] Cai JH, Chen SJ, Huang YH (2023). Clinical efficacy of tolvaptan in chronic heart failure with normal serum sodium levels. *Lingnan Journal of Emergency Medicine*.

[31] Chioncel O, Mebazaa A, Harjola VP, Coats AJ, Piepoli MF, Crespo-Leiro MG (2017). Clinical phenotypes and outcome of patients hospitalized for acute heart failure: the ESC Heart Failure Long-Term Registry. *European Journal of Heart Failure*.

[32] Novak JE, Ellison DH (2022). Diuretics in States of Volume Overload: Core Curriculum 2022. *American Journal of Kidney Diseases: the Official Journal of the National Kidney Foundation*.

[33] Ikram H, Chan W, Espiner EA, Nicholls MG (1980). Haemodynamic and hormone responses to acute and chronic frusemide therapy in congestive heart failure. *Clinical Science (London, England: 1979)*.

[34] Haldeman GA, Croft JB, Giles WH, Rashidee A (1999). Hospitalization of patients with heart failure: National Hospital Discharge Survey, 1985 to 1995. *American Heart Journal*.

[35] Schrier RW, Abraham WT (1999). Hormones and hemodynamics in heart failure. *The New England Journal of Medicine*.

[36] Maiuolo J, Oppedisano F, Gratteri S, Muscoli C, Mollace V (2016). Regulation of uric acid metabolism and excretion. *International Journal of Cardiology*.

[37] Battelli MG, Polito L, Bolognesi A (2014). Xanthine oxidoreductase in atherosclerosis pathogenesis: not only oxidative stress. *Atherosclerosis*.

[38] Cantu-Medellin N, Kelley EE (2013). Xanthine oxidoreductase-catalyzed reactive species generation: A process in critical need of reevaluation. *Redox Biology*.

[39] Richette P, Bardin T (2010). Gout. *Lancet (London, England)*.

[40] Berry CE, Hare JM (2004). Xanthine oxidoreductase and cardiovascular disease: molecular mechanisms and pathophysiological implications. *The Journal of Physiology*.

[41] Sakai H, Tsutamoto T, Tsutsui T, Tanaka T, Ishikawa C, Horie M (2006). Serum level of uric acid, partly secreted from the failing heart, is a prognostic marker in patients with congestive heart failure. *Circulation Journal: Official Journal of the Japanese Circulation Society*.

[42] Boueiz A, Damarla M, Hassoun PM (2008). Xanthine oxidoreductase in respiratory and cardiovascular disorders. *American Journal of Physiology. Lung Cellular and Molecular Physiology*.

[43] Jutabha P, Anzai N, Wempe MF, Wakui S, Endou H, Sakurai H (2011). Apical voltage-driven urate efflux transporter NPT4 in renal proximal tubule. *Nucleosides, Nucleotides & Nucleic Acids*.

[44] Hagos Y, Stein D, Ugele B, Burckhardt G, Bahn A (2007). Human renal organic anion transporter 4 operates as an asymmetric urate transporter. *Journal of the American Society of Nephrology: JASN*.

[45] El-Sheikh AAK, van den Heuvel JJMW, Koenderink JB, Russel FGM (2008). Effect of hypouricaemic and hyperuricaemic drugs on the renal urate efflux transporter, multidrug resistance protein 4. *British Journal of Pharmacology*.

[46] Butler J, Forman DE, Abraham WT, Gottlieb SS, Loh E, Massie BM (2004). Relationship between heart failure treatment and development of worsening renal function among hospitalized patients. *American Heart Journal*.

[47] Kadota M, Ise T, Yagi S, Iwase T, Akaike M, Ueno R (2016). Response Prediction and Influence of Tolvaptan in Chronic Heart Failure Patients Considering the Interaction of the Renin-Angiotensin-Aldosterone System and Arginine Vasopressin. *International Heart Journal*.

[48] Xu WZ, Wang DJ, Cao HL (2018). The effect of tolvaptan in patients with severe valvular heart disease. *Chinese Journal of Integrative Medicine on Cardio-Cerebrovascular Disease*.

[49] Zhang AT (2016). Therapeutic Evaluation for Tolvaptan to Treat Acute Myocardial Infarction Accompanied by Heart Failure. *master’s thesis*.

[50] Pollare T, Lithell H, Berne C (1989). A comparison of the effects of hydrochlorothiazide and captopril on glucose and lipid metabolism in patients with hypertension. *The New England Journal of Medicine*.

[51] Amery A, Birkenhäger W, Brixko P, Bulpitt C, Clement D, Deruyttere M (1986). Glucose intolerance during diuretic therapy in elderly hypertensive patients. A second report from the European Working Party on high blood pressure in the elderly (EWPHE). *Postgraduate Medical Journal*.

[52] Zillich AJ, Garg J, Basu S, Bakris GL, Carter BL (2006). Thiazide diuretics, potassium, and the development of diabetes: a quantitative review. *Hypertension (Dallas, Tex*.

[53] Reungjui S, Pratipanawatr T, Johnson RJ, Nakagawa T (2008). Do thiazides worsen metabolic syndrome and renal disease? The pivotal roles for hyperuricemia and hypokalemia. *Current Opinion in Nephrology and Hypertension*.

[54] Shafi T, Appel LJ, Miller ER, Klag MJ, Parekh RS (2008). Changes in serum potassium mediate thiazide-induced diabetes. *Hypertension (Dallas, Tex.: 1979)*.

[55] Prichard BN, Smith CC, Sen S, Betteridge DJ (1992). Hypertension and insulin resistance. *Journal of Cardiovascular Pharmacology*.

[56] Menon DV, Arbique D, Wang Z, Adams-Huet B, Auchus RJ, Vongpatanasin W (2009). Differential effects of chlorthalidone versus spironolactone on muscle sympathetic nerve activity in hypertensive patients. *The Journal of Clinical Endocrinology and Metabolism*.

[57] Cruickshank JM (2017). The Role of Beta-Blockers in the Treatment of Hypertension. *Advances in Experimental Medicine and Biology*.

[58] Sartori C, Trueb L, Nicod P, Scherrer U (1999). Effects of sympathectomy and nitric oxide synthase inhibition on vascular actions of insulin in humans. *Hypertension (Dallas, Tex*.

[59] Navegantes LCC, Sjöstrand M, Gudbjörnsdottir S, Strindberg L, Elam M, Lönnroth P (2003). Regulation and counterregulation of lipolysis in vivo: different roles of sympathetic activation and insulin. *The Journal of Clinical Endocrinology and Metabolism*.

[60] Jujo K, Saito K, Ishida I, Furuki Y, Kim A, Suzuki Y (2016). Randomized pilot trial comparing tolvaptan with furosemide on renal and neurohumoral effects in acute heart failure. *ESC Heart Failure*.

[61] Ames RP (1986). The effects of antihypertensive drugs on serum lipids and lipoproteins, I. Diuretics. *Drugs*.

[62] Grimm RH, Leon AS, Hunninghake DB, Lenz K, Hannan P, Blackburn H (1981). Effects of thiazide diuretics on plasma lipids and lipoproteins in mildly hypertensive patients: a double-blind controlled trial. *Annals of Internal Medicine*.

[63] Gustafson B, Hammarstedt A, Andersson CX, Smith U (2007). Inflamed adipose tissue: a culprit underlying the metabolic syndrome and atherosclerosis. *Arteriosclerosis, Thrombosis, and Vascular Biology*.

[64] Onogawa T, Sakamoto Y, Nakamura S, Nakayama S, Fujiki H, Yamamura Y (2011). Effects of tolvaptan on systemic and renal hemodynamic function in dogs with congestive heart failure. *Cardiovascular Drugs and Therapy*.

[65] Eid PS, Ibrahim DA, Zayan AH, Elrahman MMA, Shehata MAA, Kandil H (2021). Comparative effects of furosemide and other diuretics in the treatment of heart failure: a systematic review and combined meta-analysis of randomized controlled trials. *Heart Failure Reviews*.

[66] Yi JH, Shin HJ, Kim HJ (2011). V2 receptor antagonist; tolvaptan. *Electrolyte & Blood Pressure: E & BP*.

[67] Schrier RW, Gross P, Gheorghiade M, Berl T, Verbalis JG, Czerwiec FS (2006). Tolvaptan, a selective oral vasopressin V2-receptor antagonist, for hyponatremia. *The New England Journal of Medicine*.

[68] Alskaf E, Tridente A, Al-Mohammad A (2016). Tolvaptan for Heart Failure, Systematic Review and Meta-Analysis of Trials. *Journal of Cardiovascular Pharmacology*.

[69] Sen J, Chung E, McGill D (2018). Tolvaptan for Heart Failure in Chronic Kidney Disease Patients: A Systematic Review and Meta-Analysis. *Heart, Lung & Circulation*.

