# Supplementary material for: The Effect of Tolvaptan on Metabolism and Electrolyte Homeostasis in Patients with Heart Failure: A Systematic Review and Meta-Analysis
Source: Rev Cardiovasc Med. 2024 Sep 19;25(9):334. doi: 10.31083/j.rcm2509334 (PMC11440402; doi:10.31083/j.rcm2509334)
Supplement: Supplementary file 1 [file 2153-8174-25-9-334-s1.zip › 2153-8174-25-9-334-s1/RCM24692-PRISMA_2020_checklist.docx]

**PRISMA checklist.**

| **Section and Topic** | **Item #** | **Checklist item** | **Location where item is reported** |
| --- | --- | --- | --- |
| **TITLE** | | |  |
| Title | 1 | Identify the report as a systematic review.  Comment:  Title: The effect of tolvaptan on metabolism and electrolyte homeostasis in patients with heart failure: A systematic review and meta-analysis | Page 1.  Line 2-3 |
| **ABSTRACT** | | |  |
| Abstract | 2 | See the PRISMA 2020 for Abstracts checklist.  Comment:  We performed an abstract. | Page 1.  Line 5-16 |
| **INTRODUCTION** | | |  |
| Rationale | 3 | Describe the rationale for the review in the context of existing knowledge.  Comment:  The rationale is described in detail in the Introduction. | Page 1.  Line 20-41 |
| Objectives | 4 | Provide an explicit statement of the objective(s) or question(s) the review addresses.  Comment:  This study aimed to investigate the effect of tolvaptan on metabolism and electrolyte homeostasis in patients with heart failure (HF) | Page 1.  Line 36-41 |
| **METHODS** | | |  |
| Eligibility criteria | 5 | Specify the inclusion and exclusion criteria for the review and how studies were grouped for the syntheses.  Comment:  In Methods, we described in detail the study eligibility criteria (the types of studies included, the included and excluded participants, and the inclusion and exclusion criteria). | Page 2.  Line 52-62 |
| Information sources | 6 | Specify all databases, registers, websites, organisations, reference lists and other sources searched or consulted to identify studies. Specify the date when each source was last searched or consulted.  Comment:  Literature databases, such as PubMed, EMBASE, the Cochrane Library, China National Knowledge Infrastructure, VIP and WanFang Data, were systematically searched for relevant trials from inception to November 4, 2023. | Page 2.  Line 44-50 |
| Search strategy | 7 | Present the full search strategies for all databases, registers and websites, including any filters and limits used.  Comment:  The exact retrieval strategy for PubMed is listed in Supplementary file. | Supplementary file |
| Selection process | 8 | Specify the methods used to decide whether a study met the inclusion criteria of the review, including how many reviewers screened each record and each report retrieved, whether they worked independently, and if applicable, details of automation tools used in the process.  Comment:  Based on the inclusion and exclusion criteria, two authors (YX and YC) independently scrutinized articles by browsing the title, abstract and full text. Disagreements were resolved by discussion with a third author (XHZ). | Page 2.  Line 64-74 |
| Data collection process | 9 | Specify the methods used to collect data from reports, including how many reviewers collected data from each report, whether they worked independently, any processes for obtaining or confirming data from study investigators, and if applicable, details of automation tools used in the process.  Comment:  After completing the definitive inclusion of articles, data from full-text articles were extracted independently. In all stages, disagreements were solved by discussion or by consulting an independent third reviewer (XHZ). Data on the outcomes were collected and analyzed. | Page 2.  Line 64-74 |
| Data items | 10a | List and define all outcomes for which data were sought. Specify whether all results that were compatible with each outcome domain in each study were sought (e.g. for all measures, time points, analyses), and if not, the methods used to decide which results to collect.  Comment:  We have listed and defined all the results that require data in detail. | Page 2.  Line 64-74 |
|  | 10b | List and define all other variables for which data were sought (e.g. participant and intervention characteristics, funding sources). Describe any assumptions made about any missing or unclear information.  Comment:  We have listed and defined all the results that require data in detail. | Page 2.  Line 64-74 |
| Study risk of bias assessment | 11 | Specify the methods used to assess risk of bias in the included studies, including details of the tool(s) used, how many reviewers assessed each study and whether they worked independently, and if applicable, details of automation tools used in the process.  Comment:  The risk of bias and our attempt to reduce the risk of bias in the individual studies was described in the Methods section. | Page 2.  Line 76-82 |
| Effect measures | 12 | Specify for each outcome the effect measure(s) (e.g. risk ratio, mean difference) used in the synthesis or presentation of results.  Comment:  Best-evidence synthesis was described in the Methods section. This systematic review was a qualitative synthesis of the available evidence. In view of the heterogeneity of the target population, the variability of study objectives, and differences in methodological quality, a meta-analysis could not be performed. | Page 2.  Line 84-92 |
| Synthesis methods | 13a | Describe the processes used to decide which studies were eligible for each synthesis (e.g. tabulating the study intervention characteristics and comparing against the planned groups for each synthesis (item #5)).  Comment:  In Methods, we described in detail the study eligibility criteria (the types of studies included, the included and excluded participants, and the inclusion and exclusion criteria). | Page 2.  Line 52-62 |
|  | 13b | Describe any methods required to prepare the data for presentation or synthesis, such as handling of missing summary statistics, or data conversions.  Comment:  In Methods, we described in detail any methods required to prepare the data for presentation or synthesis. | Page 2.  Line 84-92 |
|  | 13c | Describe any methods used to tabulate or visually display results of individual studies and syntheses.  Comment:  In Methods, we described in detail any methods used to tabulate or visually display results of individual studies and syntheses. | Page 2.  Line 84-92 |
|  | 13d | Describe any methods used to synthesize results and provide a rationale for the choice(s). If meta-analysis was performed, describe the model(s), method(s) to identify the presence and extent of statistical heterogeneity, and software package(s) used.  Comment:  In Methods, we described in detail any methods used to synthesize results and provide a rationale for the choice(s). | Page 2.  Line 84-92 |
|  | 13e | Describe any methods used to explore possible causes of heterogeneity among study results (e.g. subgroup analysis, meta-regression).  Comment:  In Methods, we described in detail any methods used to explore possible causes of heterogeneity among study results. | Page 2.  Line 84-92 |
|  | 13f | Describe any sensitivity analyses conducted to assess robustness of the synthesized results.  Comment:  In Methods, we described in detail any sensitivity analyses conducted to assess robustness of the synthesized results. | Page 2.  Line 84-92 |
| Reporting bias assessment | 14 | Describe any methods used to assess risk of bias due to missing results in a synthesis (arising from reporting biases).  Comment:  The risk of bias and our attempt to reduce the risk of bias in the individual studies was described in both the Methods section. | Page 2.  Line 76-82 |
| Certainty assessment | 15 | Describe any methods used to assess certainty (or confidence) in the body of evidence for an outcome.  Comment:  In Methods, we described in detail any methods used to assess certainty (or confidence) in the body of evidence for an outcome. | Page 2.  Line 84-92 |
| **RESULTS** | | |  |
| Study selection | 16a | Describe the results of the search and selection process, from the number of records identified in the search to the number of studies included in the review, ideally using a flow diagram.  Comment:  Figure 1 shows in detail the flow of information through the different phases of the systematic review. | Page 2-3.  Line 95-99  Figure 1. |
|  | 16b | Cite studies that might appear to meet the inclusion criteria, but which were excluded, and explain why they were excluded.  Comment:  No applicable. |  |
| Study characteristics | 17 | Cite each included study and present its characteristics.  Comment:  Presented in the Results section and Table 1. | Page 3-4.  Line 103-113  Table 1. |
| Risk of bias in studies | 18 | Present assessments of risk of bias for each included study.  Comment:  The risk of bias and our attempt to reduce the risk of bias in the individual studies was described in the Results and in Supplementary Fig. 1. | Page 3.  Line 115-116  Supplementary Fig. 1.and Table 1 |
| Results of individual studies | 19 | For all outcomes, present, for each study: (a) summary statistics for each group (where appropriate) and (b) an effect estimate and its precision (e.g. confidence/credible interval), ideally using structured tables or plots.  Comment:  Details about the individual studies are described in the Results. | Page 3-5.  Line 103-113  Table 1. |
| Results of syntheses | 20a | For each synthesis, briefly summarise the characteristics and risk of bias among contributing studies.  Comment:  Briefly summarise the characteristics and risk of bias among contributing studies are described in Results. | Page 3-5.  Line 103-116 |
|  | 20b | Present results of all statistical syntheses conducted. If meta-analysis was done, present for each the summary estimate and its precision (e.g. confidence/credible interval) and measures of statistical heterogeneity. If comparing groups, describe the direction of the effect.  Comment:  In Methods, we described in detail above things. | Page 3-5.  Line 118-156  Fig. 2-4  Supplementary Fig. 2-3 |
|  | 20c | Present results of all investigations of possible causes of heterogeneity among study results.  Comment:  In Methods, we described in detail above things. | Page 3-5.  Line 118-156  Fig. 2-4  Supplementary Fig. 2-3 |
|  | 20d | Present results of all sensitivity analyses conducted to assess the robustness of the synthesized results.  Comment:  In Methods, we described in detail above things. | Page 5.  Line 158-160 |
| Reporting biases | 21 | Present assessments of risk of bias due to missing results (arising from reporting biases) for each synthesis assessed.  Comment:  The risk of bias and our attempt to reduce the risk of bias in the individual studies was described in the Results. | Page 5.  Line 158-160  Supplementary Fig. 4 |
| Certainty of evidence | 22 | Present assessments of certainty (or confidence) in the body of evidence for each outcome assessed.  Comment:  In Methods, we described in detail above things. | Page 3-5.  Line 118-156 |
| **DISCUSSION** | | |  |
| Discussion | 23a | Provide a general interpretation of the results in the context of other evidence.  Comment:  The main findings and their implications are described in the Discussion. The Discussion described the short the included studies, explanation of the inconsistent Results. | Page 5-8.  Line 162-244 |
|  | 23b | Discuss any limitations of the evidence included in the review.  Comment:  Limitations of the review were described in detail. | Page 8.  Line 245-256 |
|  | 23c | Discuss any limitations of the review processes used.  Comment:  Limitations of the review were described in detail. | Page 8.  Line 245-256 |
|  | 23d | Discuss implications of the results for practice, policy, and future research.  Comment:  We described above things in the conclusion of Discussion. | Page 8.  Line 258-261 |
| **OTHER INFORMATION** | | |  |
| Registration and protocol | 24a | Provide registration information for the review, including register name and registration number, or state that the review was not registered.  Comment:  The review was not registered. |  |
|  | 24b | Indicate where the review protocol can be accessed, or state that a protocol was not prepared.  Comment:  A protocol was not prepared. |  |
|  | 24c | Describe and explain any amendments to information provided at registration or in the protocol.  Comment:  No applicable. |  |
| Support | 25 | Describe sources of financial or non-financial support for the review, and the role of the funders or sponsors in the review.  Comment:  This study was supported by the Science & Technology Pillar Program in Sichuan Province, China (grant no. 2022YFS0356) and the Youth Program of National Natural Science Foundation of China (grant no. 82000418). | Funding |
| Competing interests | 26 | Declare any competing interests of review authors.  Comment:  The authors declare no conflict of interest. | Conflict of Interest |
| Availability of data, code and other materials | 27 | Report which of the following are publicly available and where they can be found: template data collection forms; data extracted from included studies; data used for all analyses; analytic code; any other materials used in the review.  Comment:  Availability of data and materials is not applicable, and and the Code availability is as well. |  |

*From:*  Page MJ, McKenzie JE, Bossuyt PM, Boutron I, Hoffmann TC, Mulrow CD, et al. The PRISMA 2020 statement: an updated guideline for reporting systematic reviews. BMJ 2021;372:n71. doi: 10.1136/bmj.n71

For more information, visit: <http://www.prisma-statement.org/>
