# Supplementary material for: The Effect of Tolvaptan on Metabolism and Electrolyte Homeostasis in Patients with Heart Failure: A Systematic Review and Meta-Analysis
Source: Rev Cardiovasc Med. 2024 Sep 19;25(9):334. doi: 10.31083/j.rcm2509334 (PMC11440402; doi:10.31083/j.rcm2509334)
Supplement: Supplementary file 1 [file 2153-8174-25-9-334-s1.zip › 2153-8174-25-9-334-s1/RCM24692-supplementary.docx]

**The exact retrieval strategy for PubMed:**

#1 "Heart Failure"[Mesh]

#2 (((((((((((((Cardiac Failure[Title/Abstract]) OR Heart Decompensation[Title/Abstract]) OR Decompensation, Heart[Title/Abstract]) OR Heart Failure, Right-Sided[Title/Abstract]) OR Heart Failure, Right Sided[Title/Abstract]) OR Right-Sided Heart Failure[Title/Abstract]) OR Right Sided Heart Failure[Title/Abstract]) OR Myocardial Failure[Title/Abstract]) OR Congestive Heart Failure[Title/Abstract]) OR Heart Failure, Congestive[Title/Abstract]) OR Heart Failure, Left-Sided[Title/Abstract]) OR Heart Failure, Left Sided[Title/Abstract]) OR Left-Sided Heart Failure[Title/Abstract]) OR Left Sided Heart Failure[Title/Abstract]

#3 #1 OR #2

#4 "Tolvaptan"[Mesh]

#5 ((((7-Chloro-5-hydroxy-1-(2-methyl-4-(2-methylbenzoylamino)benzoyl)2,3,4,5-tetrahydro-1H-1-benzazepine[Title/Abstract]) OR Samsca[Title/Abstract]) OR OPC 41061[Title/Abstract]) OR OPC-41061[Title/Abstract]) OR OPC41061[Title/Abstract]

#6 #4 OR #5

#7 ((randomized controlled trial[Publication Type]) OR randomized[Title/Abstract]) OR placebo[Title/Abstract]

#8 #3 AND #6 AND #7

**Supplemental search strategy for PubMed:**

#1 "Heart Failure"[Mesh]

#2 (((((((((((((Cardiac Failure[Title/Abstract]) OR Heart Decompensation[Title/Abstract]) OR Decompensation, Heart[Title/Abstract]) OR Heart Failure, Right-Sided[Title/Abstract]) OR Heart Failure, Right Sided[Title/Abstract]) OR Right-Sided Heart Failure[Title/Abstract]) OR Right Sided Heart Failure[Title/Abstract]) OR Myocardial Failure[Title/Abstract]) OR Congestive Heart Failure[Title/Abstract]) OR Heart Failure, Congestive[Title/Abstract]) OR Heart Failure, Left-Sided[Title/Abstract]) OR Heart Failure, Left Sided[Title/Abstract]) OR Left-Sided Heart Failure[Title/Abstract]) OR Left Sided Heart Failure[Title/Abstract]

#3 #1 OR #2

#4 "Tolvaptan"[Mesh]

#5 ((((7-Chloro-5-hydroxy-1-(2-methyl-4-(2-methylbenzoylamino)benzoyl)2,3,4,5-tetrahydro-1H-1-benzazepine[Title/Abstract]) OR Samsca[Title/Abstract]) OR OPC 41061[Title/Abstract]) OR OPC-41061[Title/Abstract]) OR OPC41061[Title/Abstract]

#6 #4 OR #5

#7 (randomized controlled trial[Publication Type])) OR (review[Publication Type])) OR (Editorial[Publication Type])) OR (meta-analysis[Publication Type])

#8 #6 NOT #7

**Table 1 The levels of serum uric acid (μmol/L) of tolvaptan and control groups before and after treatment.**

| Author, Year | Tolvaptan | |  | Control | |
| --- | --- | --- | --- | --- | --- |
|  | Before treatment | After treatment |  | Before treatment | After treatment |
| Peng YL, 2018 | 335.67±110.38 | 369.67±103.41 |  | 351.67±126.22 | 404.33±109.25 |
| Zhang D, 2016 | 544.50±112.16 | 494.93±160.79 |  | 501.64±155.75 | 526.79±85.31 |
| Cui ZT, 2023 | 400.6±146.1 | 315.6±166.2 |  | 422.4±142.5 | 403.2±217.2 |
| Ren B, 2019 | 506.63±25.43 | 418.29±26.45 |  | 502.29±31.86 | 500.92±32.20 |

Quality assessment for studies included in the present meta-analysis

**Fig. 1. Quality assessment of included RCTs.**


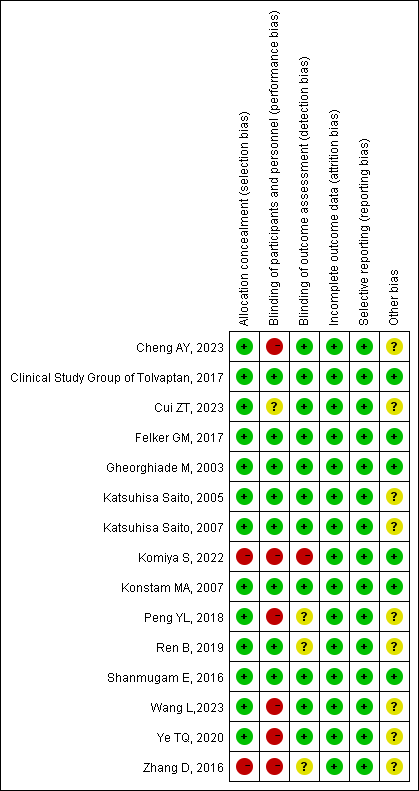

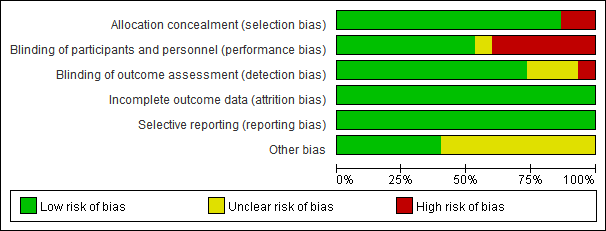


**Table 2 Newcastle-Ottawa Scale for assessing the quality of included cohort studies.**

| Author, Year | Selection | | | | Comparability on basis of design and analysis | Outcome | | | Total stars |
| --- | --- | --- | --- | --- | --- | --- | --- | --- | --- |
|  | Representativeness of exposed cohort | Selection of nonexposed cohort | Demonstration that outcome of interest was not present at start of study | Ascertainment of exposure |  | Assessment of outcome | Follow-up long enough | Adequacy of follow-up |  |
| Cai JH, 2023 | * | * | * | * | ** | * | * | * | 9 |

**
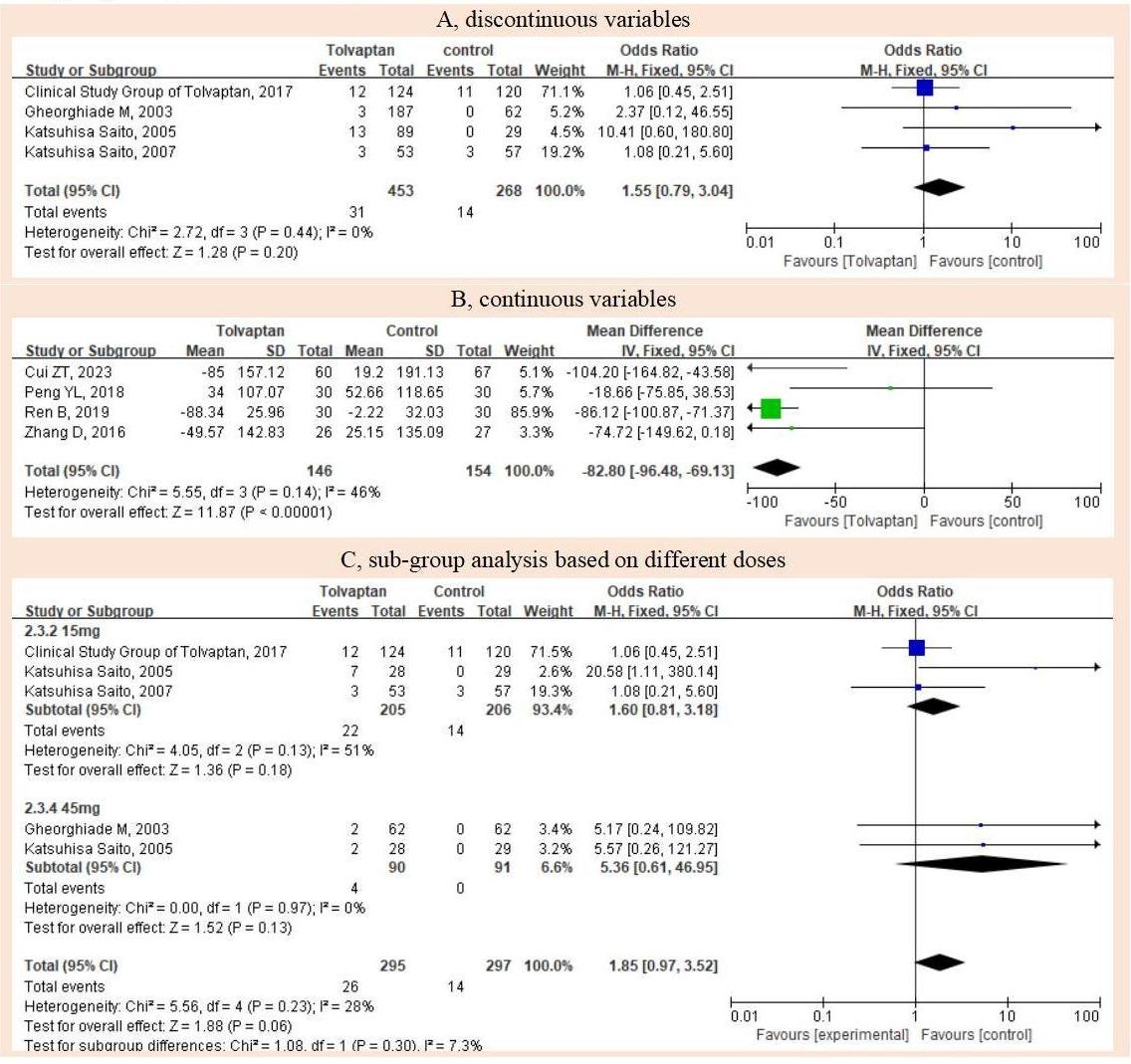
**

**Fig. 2. Forest plots after sensitivity analysis depicting the effects of tolvaptan on the incidence of hyperuricemia or increasing blood uric acid: A, discontinuous variable; B, continuous variable; C, sub-group analysis based on different doses. CI, confidence invertal; M-H, Mantel-Haenszel; IV, inverse variance.**

**
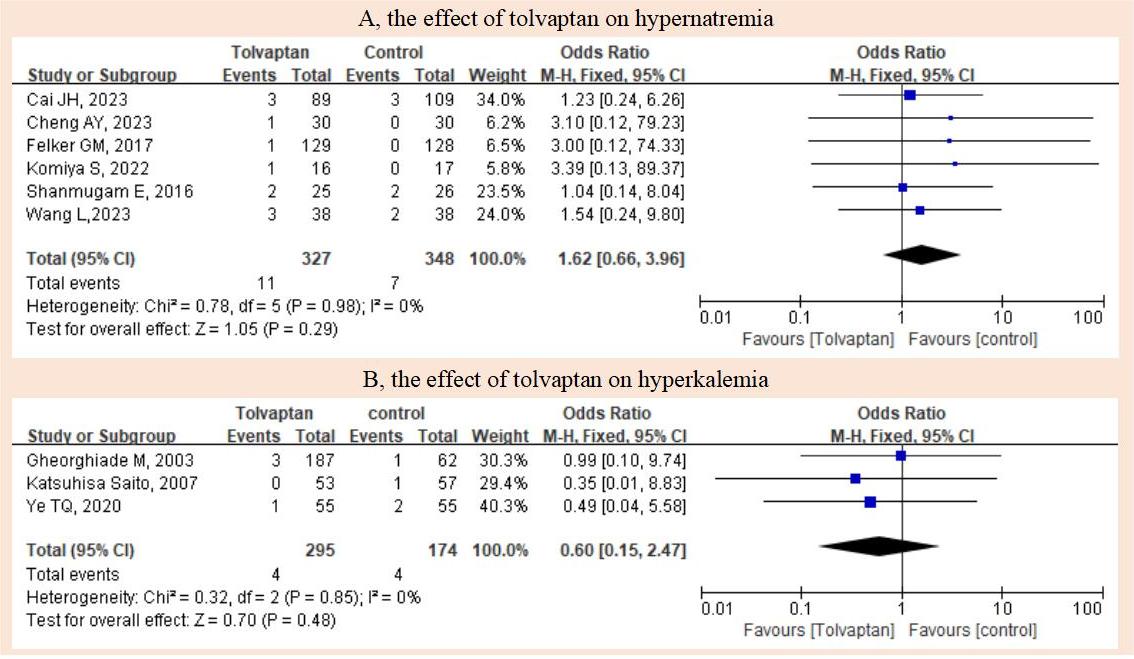
**

**Fig. 3. Forest plots after sensitivity analysis depicting the effects of tolvaptan on electrolyte: A, the effects of tolvaptan on hypernatremia; B, the effects of tolvaptan on hyperkalemia. CI, confidence invertal; M-H, Mantel-Haenszel.**


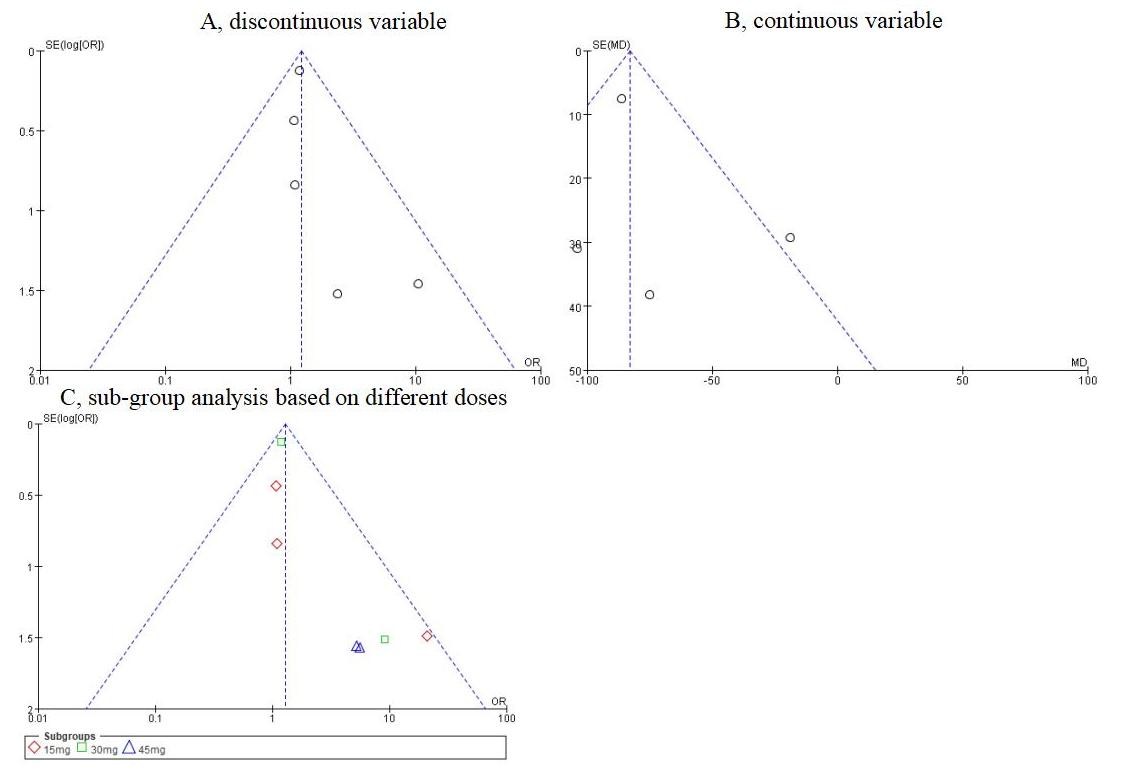


**Fig. 4. Funnel plots of comparison depicting the effects of tolvaptan on the incidence of hyperuricemia or increasing blood uric acid: A, discontinuous variable; B, continuous variable; C, sub-group analysis based on different doses. OR, odds ratio; SE, standard error.**
